# Structural Modeling of Nanobodies: A Benchmark of State-of-the-Art Artificial Intelligence Programs

**DOI:** 10.3390/molecules28103991

**Published:** 2023-05-09

**Authors:** Mario S. Valdés-Tresanco, Mario E. Valdés-Tresanco, Daiver E. Jiménez-Gutiérrez, Ernesto Moreno

**Affiliations:** 1 Faculty of Basic Sciences, University of Medellin, Medellin 050026, Colombia; daiverjimenez05@gmail.com; 2 Centre for Molecular Simulations and Department of Biological Sciences, University of Calgary, Calgary, AB T2N 1N4, Canada; mario.valdeztresanco@ucalgary.ca

**Keywords:** artificial intelligence, protein structure, protein modeling, nanobody, antibody

## Abstract

The number of applications for nanobodies is steadily expanding, positioning these molecules as fast-growing biologic products in the biotechnology market. Several of their applications require protein engineering, which in turn would greatly benefit from having a reliable structural model of the nanobody of interest. However, as with antibodies, the structural modeling of nanobodies is still a challenge. With the rise of artificial intelligence (AI), several methods have been developed in recent years that attempt to solve the problem of protein modeling. In this study, we have compared the performance in nanobody modeling of several state-of-the-art AI-based programs, either designed for general protein modeling, such as AlphaFold2, OmegaFold, ESMFold, and Yang-Server, or specifically designed for antibody modeling, such as IgFold, and Nanonet. While all these programs performed rather well in constructing the nanobody framework and CDRs 1 and 2, modeling CDR3 still represents a big challenge. Interestingly, tailoring an AI method for antibody modeling does not necessarily translate into better results for nanobodies.

## 1. Introduction

Nanobodies (Nbs) are the single binding domains of camelid heavy chain antibodies. Structurally, they share similarities with the variable heavy chain domain (VH) of traditional antibodies, consisting of a highly conserved region called framework and the antigen recognition region formed by three hypervariable loops, also called complementarity determining regions (CDRs) [1]. Nbs are much smaller (only 15 kDa) than human antibodies and their derivatives, but nonetheless can achieve similar affinities. Furthermore, they are highly stable and easy to produce [2,3]. These characteristics have positioned them as fast-growing biologic products in the biotechnology market.

The number of applications for Nbs is expanding steadily [3,4,5,6,7,8]. Several of these applications require protein engineering, which in turn would greatly benefit from having a reliable three-dimensional (3D) model of the Nb being modified [9,10,11]. However, as with antibodies, structural modeling of Nbs is still a challenge [12,13]. There are several hundreds of Nb crystallographic structures deposited in the Protein Data Bank (PDB) [14,15]; however, this is still insufficient to represent the huge structural and sequence variability found in Nb hypervariable loops. Furthermore, the CDR3 in Nbs shows a spectrum of conformations, lengths, and sequence variability greater than that of antibodies [16], which increases the difficulty for modeling their 3D structure. Nonetheless, homology modeling of nanobodies have been attempted for practical purposes, as in [17,18], and the recent developments in artificial intelligence (AI) methods for protein modeling, which have outperformed conventional methods [19,20,21], have been applied also to the modeling of antibodies and nanobodies [12,13,22,23,24,25,26,27].

Several AI methods have been developed in recent years to tackle the problem of protein modeling [28,29,30]. In this scenario, the development of AlphaFold represented a revolution in high-accuracy 3D protein modeling [31]. Since then, several methods have come to light, improving aspects, such as speed, computational resource consumption, and modeling accuracy [32]. AI programs especially designed to model complete antibodies and their fragments have been generated, including an AI model—Nanonet [12]—designed for modeling Nb structures. Given the similarity between Nbs and antibody VH domains, all AI models developed for antibodies can in principle be used for Nb modeling [13,26,27]. So far, however, AI methods have not been extensively tested for nanobody modeling. In this regard, comparing their modeling efficiency for this important class of proteins is important to establish to which extent the constructed models can be considered reliable for different practical applications, and as a guide for researchers in selecting the most appropriate modeling programs.

In this study, we have compared the performance in Nb modeling of six state-of-the-art AI-based programs, either designed for general protein modeling, such as AlphaFold2 [31], OmegaFold [33], ESMFold [34], and trRosetta (Yang-Server in the most recent Critical Assessment of Structure Prediction competition—CASP15) [28] or specifically designed for antibody modeling, such as IgFold [13] and Nanonet [12]. Interestingly, tailoring an AI program for antibody modeling does not necessarily translate into better results for nanobodies.

## 2. Results and Discussion

### 2.1. Dataset Selection and Validation

**Figure 1:**
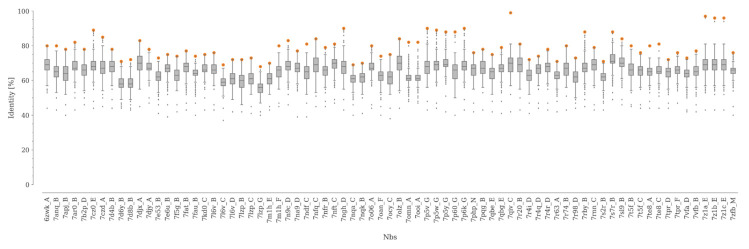
Sequence identity between each Nb in the dataset and the rest of the Nbs in the SAbDaB database. The sequence identity distributions are represented with boxplots. The lower and upper edges of the box represent the first (Q1) and third quartile (Q3), respectively. The difference Q3–Q1 is known as the interquartile range (IQR). Whiskers extend to the minimum and maximum points within ±1.5 × IQR, respectively. The maximum value of sequence identity for each distribution is represented as an orange dot.

For this study, we built a curated, non-redundant dataset of Nbs, none of which had been included in any of the training sets of the benchmarked programs. Following the procedure described above, we obtained a dataset of 75 unique Nbs with a median resolution of 2.59 Å (Figure 1, Table S1 [Supplementary Information (SI)], Figure S1 [SI]).

The median sequence identity between the Nbs and the rest of the structures not contained in our dataset was between 56 and 71% (Figure 1). On the other hand, the maximum value of sequence identity within our dataset is below 90% in 91% of the cases, with only four pairs of Nbs showing a sequence identity higher than 95% (Figure S2 [SI]). Here, it is worth noting that for Nbs, as well as for antibodies, even point mutations can induce important structural changes in CDR3 [35].

### 2.2. Structure Prediction Accuracy

**Figure 2:**
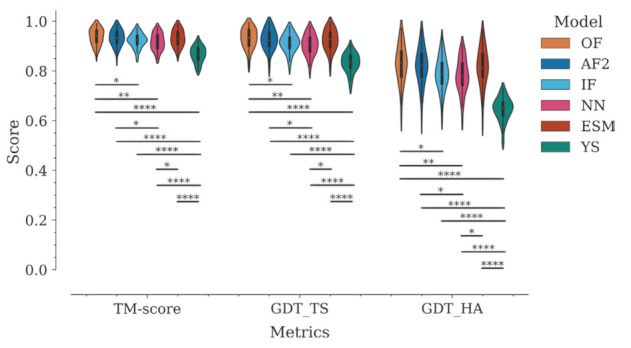
Assessment of the modeling accuracy of the six AI programs using global superposition metrics (TM-score, GDT-TS, and GDT-HA). The distributions of metric values are represented with violin plots, which combine a kernel density plot (outer) to show the distribution of values and a boxplot (inner) that summarizes the distribution statistics. In the boxplot, a white dot represents the median, the thick gray bar in the center represents the interquartile range, and the thin gray line accounts for the rest of the distribution. Statistical significances are represented with asterisks according to the following convention: * *p* ≤ 0.05, ** *p* ≤ 0.01, *** *p* ≤ 0.001, and **** *p* ≤ 0.0001.

We compared the performance of six AI models for 3D structure prediction of Nbs: OmegaFold (OF), AlphaFold2 (AF2), IgFold (IF), NanoNet (NN), ESMFold (ESM), and trRosetta (referred to as Yang-Server in the latest CASP15 and herein) (YS). Modeling accuracy was initially evaluated using global superposition structural similarity metrics—TM-score, GDT_TS, and GDT_HA—traditionally used in CASP competitions. Figure 2 shows the distribution of values by program for each metric.

In general, all tested programs performed well according to these global metrics. The Yang-Server showed the most discrete performance with medians of 0.87, 0.84, and 0.65 for TM-score, GDT_TS, and GDT_HA, respectively. On the other hand, OmegaFold, AlphaFold2, ESMFold, IgFold, and NanoNet, in decreasing order, showed medians above 0.91 for TM-score and GDT_TS, respectively, and above 0.78 for GDT_HA (Figure 2, Table S2 [SI]).

**Figure 3:**
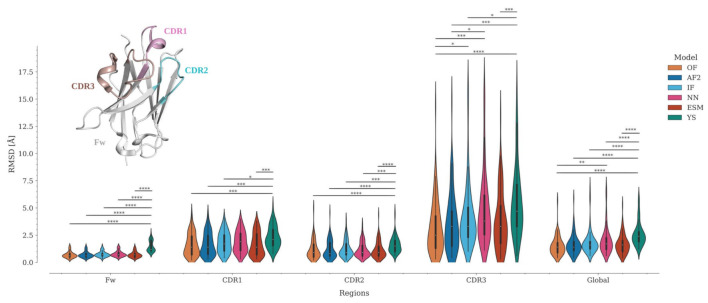
Assessment of modeling accuracy by RMSD for the Fw and CDR regions, for OmegaFold, AlphaFold2, IgFold, Nanonet, ESMFold and Yang-Server. RMSD distributions are represented using violin plots. Nb regions are colored as follows: Framework (Fw) as gray; CDR1—pink, CDR2—cyan, and CDR3—brown. Statistical significances: * *p* ≤ 0.05, ** *p* ≤ 0.01, *** *p* ≤ 0.001, and **** *p* ≤ 0.0001.

TM-score and GDT_TS estimate the percent structural similarity between the model and the experimental structure. Values above 0.5 indicate that both structures have the same folding, while values above 0.9 indicate that they are structurally identical [36]. However, unlike other protein families, antibodies and Nbs present a major challenge for modeling techniques due to their CDRs. The framework is modeled correctly in most cases due to the high conservation of this region, whereas most of the modeling errors are concentrated in the CDRs, especially in CDR3. This fact generates an important bias in the metrics. This can be reflected in the variation of the global RMSD compared to per-region RMSDs (Figure 3, Table S2 [SI]). To objectively evaluate the modeling accuracy of each program, we divided the Nbs into four regions—Framework (Fw), CDR1, CDR2, and CDR3—and calculated the RMSD for each of them (Figure 3, Table S2 [SI]).

Because of the high conservation of the immunoglobulin domain framework, it is expected that all programs should correctly predict the structure of this region. Interestingly, while OmegaFold, AlphaFold2, IgFold, Nanonet, and ESMFold predicted the Fw structure with high accuracy (0.6 ≤ RMSD median ≤ 0.7), the Yang-Server yielded more discrete results (RMSD median = 1.2). In fact, only the Yang-Server shows significant statistical differences with respect to the other programs (Figure 3, Table S2 [SI]). Modeling of CDRs, in contrast, poses a challenge for all programs. CDR2 has predicted more accurately (0.8 ≤ median RMSD ≤ 1.5) than the other CDRs, with only a few structures showing RMSD values above 2.5 Å (Figure 3, Table S3 [SI]) and significant differences only for the Yang-Server (Figure 3, Table S2 [SI]). CDR1 predictions remain in an acceptable range (1.4 ≤ median RMSD ≤ 2.1) with an increase in the number of structures with RMSD > 2.5 Å (Figure 3, Table S3 [SI]), but without considerable significant statistical differences among them, except for the Yang-Server with respect to all but Nanonet.

CDR3 predictions, on the other hand, are the most inaccurate (2.5 ≤ median RMSD ≤ 4.7), with about or more than 50% of the structures showing RMSD > 2.5 Å (Figure 3, Table S3 [SI]). Significant statistical differences among several programs (Yang-Server, IgFold, and Nanonet) are observed for this region. OmegaFold, with a median RMSD of 2.5 Å, performs well in both overall value and RMSD per region, followed by AlphaFold2, IgFold, and ESMFold (median RMSD = 3.3 Å for CDR3), Nanonet (median RMSD = 3.8 Å) and finally Yang-Server (median RMSD = 4.7 Å)

### 2.3. Structure Prediction Accuracy by Sequence Position

**Figure 4:**
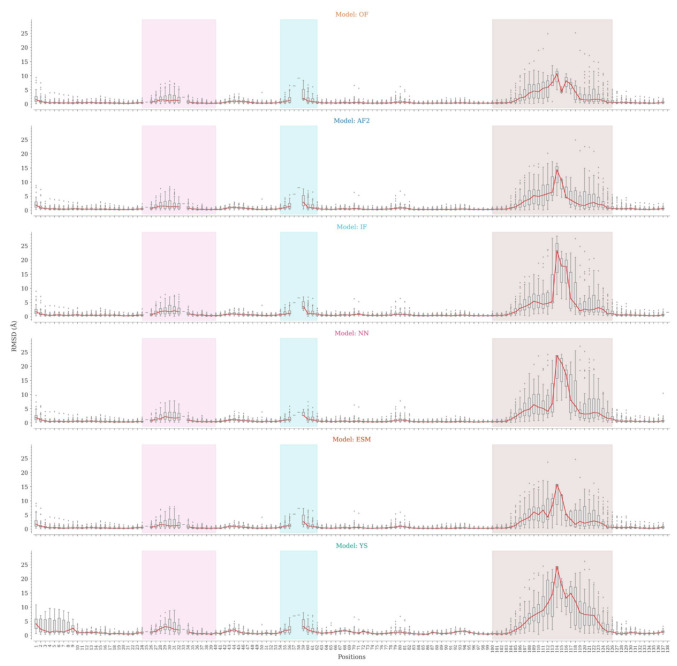
Distribution of Cα RMSD values by position for the OmegaFold, AlphaFold2, IgFold, Nanonet, ESMFold, and Yang-Server models. CDR1, CDR2, and CDR3 regions are colored pink, cyan, and brown, respectively. The RMSD distributions are represented by boxplots.

As shown above, global superposition metrics are not suitable for estimating the accuracy of Nb modeling due to their structural characteristics. At the sequence region level, we observed a considerable variation in the accuracy of CDR modeling, especially for CDR3. We then analyzed the structures generated by the tested programs at the sequence position level to identify the regions that mark the differences in modeling. For each sequence position, we compared the RMSD values for Cα atoms and the whole amino acids between the predicted and experimental structures (Figure 4).

All programs, except the Yang-Server, are consistent regarding framework modeling, with slight variations in the N-terminal region and non-CDR loops. The Yang-Server shows slight structural variations in the whole framework as compared to the rest of the programs, while a greater variation is observed in the N-terminal segment. NanoNet uses Modeller, while IgFold and the Yang-Server use Rosetta for side-chain modeling. NanoNet shows considerable RMSD variations when all heavy atoms are considered, followed by IgFold and Yang-Server with fewer variations (Figure S3 [SI]). The side chains in the framework region are consistently well-modeled by OmegaFold, AlphaFold2, and ESMFold (Figure S3). The results for CDR1 are similar in all cases, with minor differences and slightly higher medians for Yang-Server. On the other hand, CDR2 shows appreciable variations. Positions 57, 58, and 59 are poorly represented in the dataset, with less than five structures having amino acids at these positions (Figure S4 [SI]). NanoNet slightly outperformed the rest of the programs.

Finally, the main differences were found for CDR3 modeling. The lowest RMSD distributions by position were achieved by OmegaFold, followed by AlphaFold2, IgFold, ESMFold, Nanonet, and Yang-Server. Except for Yang-Server, the differences are relatively small for short CDR3s, becoming more accentuated for Nbs with the longest loops. The C-terminal segment of CDR3 shows the lowest variations, probably associated with the frequent formation of secondary structure elements in this region, while the N-terminal part shows more discrete results. Nonetheless, in both cases the structural variations are considerable. 

The observed differences in modeling performance can be due to the intrinsic characteristics of each AI model and the representation and structural variability of the Nbs with different CDR3 lengths in their training sets. OmegaFold does not require a multiple sequence alignment (MSA), instead using a new combination of a large pre-trained language model for sequence modeling and a geometry-inspired transformer model for structure prediction. According to its authors, this allows the modeling of orphan proteins and antibodies from their amino acid sequences [33]. Similarly, ESMFold is based on ESM-2 (Evolutionary Scale Model), which is a language model that internalizes evolutionary patterns linked to structure, eliminating the need for external evolutionary databases, MSAs, and templates [34]. IgFold and NanoNet do not require either a multiple sequence alignment. IgFold and NanoNet were trained to reproduce antibody and Nb structures, which limits the generation of atypical structures and therefore unrepresented in their training sets [12,13].

AlphaFold2, on the other hand, predicts the structure from neural networks and training procedures based on evolutionary, physical, and geometrical constraints of protein structures. To do so, it requires the protein primary sequence and a multiple sequence alignment, therefore, sequence identity and coverage of the different regions are crucial to obtain an accurate model [31]. Yang-Server also requires an MSA and, in most cases, including a homologous template yields better modeling results [37]. Given the number of available structures and the spectrum of lengths, composition, and conformations of CDR3, it is difficult to generate an MSA for Nbs with full coverage of their sequences. However, general protein modeling programs such as OmegaFold, AlphaFold2, and ESMFold, have been exposed to a wide and diverse set of protein structures, which may explain their better results in modeling CDRs, especially CDR3. (Figure 4).

### 2.4. CDR3 Structure Prediction Accuracy

**Figure 5:**
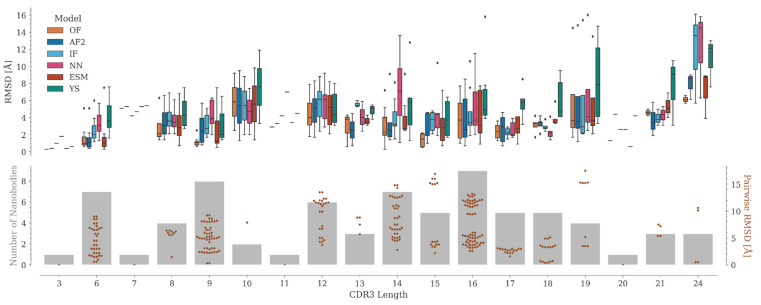
RMSD distributions per CDR3 length. The upper panel shows the RMSD distributions (as boxplots, with outliers as black diamonds) per CDR3 length for OmegaFold, AlphaFold2, IgFold, Nanonet, ESMFold, and Yang-Server. The lower panel shows the number of Nbs in the dataset per CDR3 length (bars in grey) and the pairwise RMSD values among CDR3s of the same length (swarm plot in brown).

The accuracy of CDR3 modeling depends mainly on its length (Figure 5). Several CDR3 lengths are poorly represented in our dataset, where the number of Nbs varies from one (for lengths 3, 7, 11, and 20) to a maximum of nine (for length 16).

Depending on the AI model, the median RMSD of the predictions varies along the CDR3 length range. In most cases, OmegaFold achieved the best predictions, followed by AlphaFold2, ESMFold, IgFold, Nanonet, and lastly, Yang-Server. Although no direct correlation between CDR3 length and RMSD values is observed among the experimental structures, the structural variability might influence the predictions. For example, for length 15, where the structural variation in CDR3 is considerable, the predictions are relatively consistent, especially for OmegaFold, which yields RMSD values all below 2 Å (Figure 5). This is probably because this length is the most represented in the PDB and, therefore, in the training sets of the tested programs (Figure S5 [SI]). On the other hand, CDR3s with lengths 17 and 18 adopt a similar conformation, hence the RMSD between the structures is relatively small and their modeling is consistently good for all the tested programs, except for the Yang-Server.

For lengths 19 and 24, a few models with high RMSD are generated. For these particular cases, the experimental structure has marked differences from the rest of the Nbs with the same CDR3 length. In 7tpr_D [38] (length 19), the antigen is positioned in-between CDR3 and the framework, thus altering the common CDR3 conformation (Figure S6 [SI]). For length 24, the 7d8b_B and 7d6y_B structures [39] correspond to an engineered human variable heavy chain domain. These Nbs do not have a canonical disulfide bond and show two alpha helix segments in CDR3, which causes the N-terminal portion of this region to be displaced with respect to the rest of the structures of the same length (Figure S6 [SI]). Interestingly, although they differ in only two amino acids and have similar structures, for 7d6y_B, unlike 7d8b_B, a significant improvement was obtained when modeled with its antigen (see Section 2.6.2) (Figure S10 [SI]). However, in both cases, it was not possible to correctly reproduce the secondary structure motifs present in CDR3, probably because of the poor representation of this CDR3 length in the available structures (Figure S5 [SI]).

### 2.5. Nanobody Modeling Confidence

The confidence value is an important metric in protein structure modeling that allows to estimate how reliable a model can be considered. NanoNet does not produce any metric to estimate its modeling confidence. OmegaFold, AlphaFold2, ESMFold, and Yang-Server do offer a measure of confidence called pLDDT (predicted local distance difference test) on a 0–100 scale, which corresponds to the predicted model score of the lDDT-Cα metric [31,33]. IgFold, on the other hand, offers an error estimate based on per-residue Cα deviations [13]. These metrics differ both conceptually and in scale. Typically, a pLDDT above 90 indicates a highly reliable model, 70 < pLDDT < 90 is considered reliable, while a model with pLDDT below 70 should be carefully reviewed. In contrast, there is not an established RMSD value below which a model is defined as reliable, although in practice, protein models with global RMSD below 4 Å are considered good. 

**Figure 6:**
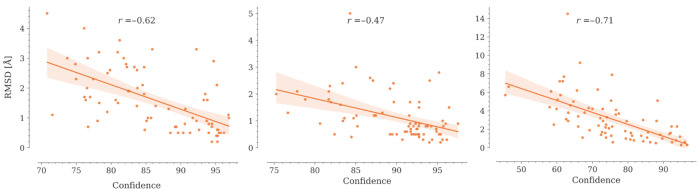
Correlation between the RMSD values and the average predicted confidences by OmegaFold for the CDR regions in the 75 Nbs conforming to our dataset. Regression lines are shown in orange. Translucent bands around the regression lines indicate the 95% confidence interval for the regression estimates. Spearman correlation coefficients (r) are shown in the graphs. In all cases, the *p*-value < 0.05.

AlphaFold2 and OmegaFold report values of pLDDT below 70 for predicted CDR3s, which correlates with the RMSD values obtained for these loops between the models and their crystallographic structures (Figure 6 and Figure S7 [SI], Table S4 [SI]). Yang-Server shows the lowest correlation, while OmegaFold achieves the highest.

Although the obtained correlation coefficients are significant, it is not possible to establish a priori whether a model is reliable or not. Since CDR3 is the region that interacts more frequently with the antigen, further studies are required to estimate whether the generated model can be used for bioinformatics approaches that demand high structural accuracy, such as protein–protein docking.

### 2.6. Structure Prediction Accuracy Varying Modeling Parameters

#### 2.6.1. Number of Recycles

Among the tested programs, only AlphaFold2 and OmegaFold allow parameter modification, specifically the number of recycles, which controls the degree of structural model refinement. In several cases, AlphaFold2 has been shown to improve the prediction of disordered structures or de novo proteins by increasing the number of recycles [40]. OmegaFold has an equivalent tunable parameter, although its functionality has not been extensively assessed yet. Here, we tested several values for the number of recycles to assess their effect on the modeling of different Nb regions.

The models generated with AlphaFold2 using ten recycles slightly improved the predictions for CDR1 and CDR3, while slightly worsening those for CDR2. No considerable variations were observed for the framework and global modeling. In all cases, there were no statistically significant differences (*p*-value > 0.05). On the other hand, using 20 recycles with OmegaFold does not translate into any considerable variation for any Nb region. Interestingly, using four recycles slightly improves CDR1 and CDR2 predictions, while losing accuracy in CDR3 modeling. However, statistically, there are no significant differences in any case (Figure S8 [SI]). Based on these results, using four recycles instead of the default value (number of recycles = 10) might be preferable since it decreases the computational time (see below).

#### 2.6.2. Modeling Nanobodies in Complex with Their Antigens with AlphaFold-Multimer

Currently, we are lacking enough Nb structures to estimate the effect of antigen binding on CDR conformations. Interestingly, there are a few cases where the same Nb shows several conformations, even in the free state (Figure S9 [SI]). In other cases, the structural variations between Nbs with the same CDR3 might be attributed to the formation of an Nb-antigen complex. Most of the Nbs used in the parameterization of AI models are complex with their antigens, thus making it difficult to determine whether the observed conformations would remain the same in their free states. 

Alphafold2 can model single chains with high reliability; however, it may fail in predicting protein structures in the context of certain complexes [41]. Using AlphaFold-multimer, we tested whether there is an improvement in CDR3 modeling for Nbs complexed with their antigens. To perform this analysis, we selected 41 structures considering the size and complexity of the antigen (Table S5 [SI]). The results from these calculations were mixed. In several cases (7nfr_B, 7t5f_B, 7m1h_E, 7olz_B, 7rby_B, and 7d6y_B) significant improvements were achieved, while in other cases (7php_N, 7zfb_M, 7pqg_B, and 7e53_B) the program produced significantly worse results. In all other cases, regardless of CDR3 length, the results are similar to those obtained for the free Nb (Figure S10 [SI]).

#### 2.6.3. Energy Minimization

Commonly, energy minimization is used to remove clashes among atoms in the structure. However, this does not imply a significant improvement in the models since such geometry optimization does not significantly change the overall conformation of loops and other regions [31]. Here, we applied energy minimization to all the generated models. The results show that, indeed, there are no significant improvements (Figure S11 [SI]).

### 2.7. Computation Time

**Figure 7:**
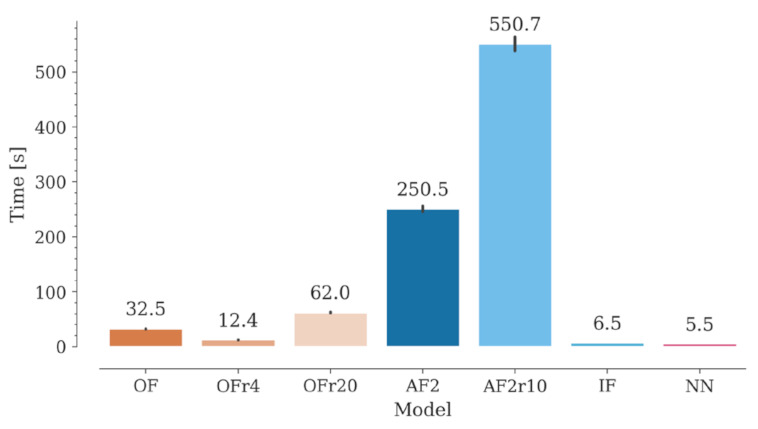
Computation time for the generation of a structural Nb model with OmegaFold, AlphaFold2, IgFold, and Nanonet. Computation times for OmegaFold and AlphaFold with different recycle numbers are also included.

Nb libraries may contain billions of sequences, with many possible different structures. In recent approaches, library design seeks to favor structures with certain CDR3 geometries (e.g., concave, or convex) that will presumably bind to specific antigens [42,43]. With the increasing development of synthetic libraries [44], methods for reliable estimation of the CDR structural diversity would be of great value for in silico design of Nb libraries with desired conformational properties. Along with accuracy, computational time becomes an important factor to be considered when modeling such a high number of structures. In this context, NanoNet takes the lead, followed by IgFold, OmegaFold, and lastly, AlphaFold2 (Figure 7). ESMFold was used in this study through the ESM Metagenomic Atlas API (application programming interface), while the Yang-Server was used through its dedicated server (https://yanglab.nankai.edu.cn/trRosetta/, accessed on 1 May 2023). ESMFold is extremely fast, obtaining results in approximately one second. However, this may depend on the demand on the server, so it might have limitations in the number of requests. Yang-Server modeling can take approximately one hour due to the algorithm and server capacity (only 30 active jobs at a time). In both cases, however, it is possible to install a standalone version for local use.

For OmegaFold, the computation time improves when decreasing the number of recycles from ten (default) to four, without affecting its accuracy. On the other hand, we found that increasing the number of recycles beyond the default value drastically increases the computational time without any noticeable benefit in modeling accuracy for both OmegaFold and AlphaFold2. It is worth noting that NanoNet may include sidechain modeling with Modeller, which would increase the computational time by a factor of 170–900 approximately, depending on the number of sequences being simultaneously processed. Finally, energy minimization not only does not improve modeling results, but it also adds computation time. The extra time required varied between 10 and 50 s per structure using our hardware configuration.

## 3. Materials and Methods

### 3.1. Benchmark Dataset

We started from the SAbDaB database [45], containing a total of 981 structures as of 15 June 2022. Firstly, we removed the PDB structures used for the parameterization of the AI programs to be compared. Next, incomplete structures and duplicated Nbs, identified from a pairwise comparative analysis of their amino acid sequences using Blastp [46,47,48], were withdrawn. For the subsequent analyses, the sequences were numbered according to Aho’s scheme using ANARCI [49]. All modeling was carried out from the primary structure of the Nb, without using templates, except for the modeling of Nbs in complex with their antigens, where the crystallographic structure of the antigen was used as a template.

### 3.2. Artificial Intelligence Models

Currently, AI methods have reached a high level of precision in protein modeling, as evidenced in the latest CASP competitions, where the first positions have been occupied by robust AI-based models (https://predictioncenter.org/index.cgi, accessed on 1 May 2023). The number of these AI protein modeling programs is rapidly increasing, making it difficult to perform comprehensive benchmarking. For this study, we selected six AI modeling programs that have stood out for their performance in general protein modeling and/or antibody modeling.

The first choice was AlphaFold2 [31], which has become a gold standard in protein modeling, inspiring the development of other AI methods. Further, we selected OmegaFold [33] and ESMFold [34], which are based on protein language models and therefore, by difference with AlphaFold2, do not involve the generation of multiple sequence alignments. As reported by its authors, OmegaFold’s results are comparable to those of AlphaFold2 for proteins in general and are better for orphan proteins and antibodies [33]. ESMFold is based on ESM-2, which in a study conducted by its authors outperformed all single-sequence protein language models tested in a variety of structure prediction tasks [34]. ESMFold has gained popularity with the recent release of the ESM Metagenomic Atlas (https://esmatlas.com, accessed on 1 May 2023) that incorporates an application programming interface (API) to perform protein modeling easily and quickly. The fourth program chosen for our study is the Yang-Server [37,50], which finished as the top-ranked program in the most recent CASP competition (CASP15, https://predictioncenter.org/casp15/zscores_final.cgi, accessed on 1 May 2023). Finally, we included two programs—IgFold [13] and Nanonet [12]—that were specifically designed for antibody modeling and have proven to be considerably better than conventional homology modeling methods [13,26,33]. Below we provide a brief description of each of these programs and their use in this study.

#### 3.2.1. AlphaFold2

AlphaFold2 is an AI model developed by DeepMind that incorporates a neural network architecture and training procedures based on evolutionary, physical, and geometrical constraints of protein structures [31]. The AlphaFold network directly predicts the 3D coordinates of all heavy atoms for a given protein using as input the primary amino acid sequence and aligned sequences of homologs.

AlphaFold2 is composed mainly of two blocks: (1) the sequence information module, and (2) the structure module, both based on transformers. The first module, called Evoformer, processes the input and generates a multiple sequence alignment (MSA) and a residue pair matrix. The main innovation in the Evoformer block is the mechanisms to exchange information within the MSA and pair representations, that enable direct reasoning on the spatial and evolutionary relationships. The second module generates the 3D structure using the pair representation and the single representation of the MSA, with a mechanism that allows simultaneous local refinement of all parts of the structure, reasoning about unrepresented side chain atoms, and weighing the correct residue orientations. After an initial structure is generated, an interactive recycling process is carried out that reuses the entire network to obtain a refined final structure [31]. AlphaFold2 was trained using the PDB and PDB70 for template search and over millions of protein families’ sequences using Uniref90, BFD, Uniclust, and MGnify for the MSA construction. At CASP14, AlphaFold was the top-ranked protein structure prediction method [51].

ColabFold, on the other hand, offers a user-friendly and fast implementation of AlphaFold2 [40]. In this application, an MSA is generated with MMseqs2 [52,53], simplifying the process and reducing the computation time. For this study, we used localColabFold v1.4.0 to run the calculations on our computers (https://github.com/YoshitakaMo/localcolabfold, accessed on 1 May 2023). For Nbs in complex with an antigen, we employed AlphaFold-multimer [41] as implemented in ColabFold using the free Google Colab service. In these calculations, we kept the default AlphaFold-multimer parameters, while for modeling Nbs in the free state we used the AlphaFold2 default configuration (three recycles), as well as ten recycles.

#### 3.2.2. OmegaFold

OmegaFold was the first computational method to successfully predict high-resolution protein structure from its single primary sequence alone [33]. It uses OmegaPLM, a deep transformer-based protein language model, to learn single- and pairwise-residue embeddings (or representations) as powerful features that model the distribution of sequences. These embeddings are fed into Geoformer, a geometry-inspired transformer neural network, to distill the structural and physical pairwise relationships between amino acids. Lastly, a structural module predicts the 3D coordinates of all heavy atoms. OmegaFold shares similarities with AlphaFold2, in their first stage of extracting per-residue pair representation information and a second stage of generating the three-dimensional structure from this representation. However, they also have notable differences. OmegaFold, by incorporating OmegaPLM, which captures structural and functional information encoded in the amino-acid sequences through the embeddings, does not require multiple sequence alignment, and the Geoformer has a focus primarily on vector geometry as opposed to the evolutionary variation of the AlphaFold2’s Evoformer. The full model was jointly trained on ~110,000 single-chain structures from the PDB and all single domains from the SCOP v1.75 database with at most 40% sequence identity. According to the authors, in several cases, OmegaFold achieves the same or better precision than RoseTTaFold and AlphaFold2, particularly for orphan proteins and antibodies [33]. Here, we used the default configuration (number of recycles = 10) and tried two other values for this parameter: 4 and 20.

#### 3.2.3. ESMFold

ESMFold [34] is an AI model for protein structure prediction that shares characteristics with AlphaFold2 [31] and OmegaFold [33]. Like OmegaFold, it uses a powerful protein language model called ESM-2 (evolutionary scale model) to process the input [34]. This model is the improved version of ESM-1b [54] (used as a reference by OmegaPLM as well), with a large number of parameters, which internalizes evolutionary patterns linked to the structure from sequences, eliminating the need for external evolutionary databases, multiple sequence alignments and templates. ESMFold uses a simple architecture that takes advantage of evolutionary information captured by the ESM-2 language model. The architecture is divided into two parts, similar to AlphaFold2. The first part is a folding module that takes the features of the language model as input and produces representations using a simplified version of AlphaFold’s Evoformer. The second part is the structure module similar to AlphaFold, which generates 3D atomic coordinates from those representations. About 60 million Uniref50 [55] protein sequences were used for ESM-2 training. For its part, ESMFold was trained with selected PDB structures using the same procedure described for AlphaFold. Additionally, they incorporated around 13 million structures generated by AlphaFold with mean pLDDT > 70 [34]. For this work, we used the ESMFold API available in the ESM Metagenomic Atlas (https://esmatlas.com/, last accessed on 23 April 2023).

#### 3.2.4. Yang-Server

The Yang-Server is a recent implementation, with several improvements, of trRosetta (transform-restrained Rosetta) [37]. Initially, the trRosetta method was inspired by other algorithms, such as RaptorX [24] and the first version of AlphaFold [56], for distance and contact prediction. Similar to these methods, it uses an MSA as input in the first step, and using a deep residual convolutional network, predicts the distance, contact, and orientation matrices of all pairs of residues in the protein. In the second step, a constrained minimization-based fastRosetta model construction protocol with distance and orientation constraints derived from the network outputs is carried out. The predicted geometries are then transformed into restraints to guide the structure prediction by direct energy minimization, which is implemented under the Rosetta framework [50]. The trRosetta version implemented in the Yang-Server has several improvements, including the MSA generation and selection improvements, a new neural network architecture for the distance and orientation prediction between residues, and the inclusion of template-based constraints [37]. Unlike the previously discussed methods, the Yang-Server was trained with just over 16,000 high-quality structures (≤2.5 Å) but using a robust MSA selection method from five alternatives based on different sources, ensuring sufficient sequence representation in the MSA [50]. As mentioned above, Yang-Server was the top-ranked program in the recent CASP15 competition.

#### 3.2.5. IgFold

IgFold uses a principle similar to ESMFold but is specifically applied to antibodies. The input is processed by AntiBERTy [23], a transformer language model pre-trained on natural antibody sequences, similar to ESM-2 or OmegaPLM. This model extracts representations of all residues from the protein sequence without requiring an MSA. These representations are then processed by the structure module, which uses a modified version of the one implemented in AlphaFold2, to generate the 3D coordinates of the model’s backbone. Finally, PyRosetta [57] is used to generate the side chains of all residues and obtain the final model [13]. The AntiBERTy model was trained using 558 million antibody sequences [23]. IgFold was trained using about 4300 and 37,000 structures from SAbDab and those modeled by AlphaFold, respectively, consisting of paired and unpaired sequences, including nanobodies. This AI model showed better results than other previously proposed models [13] such as AbodyBuilder [58], DeepAb [26], and Ablooper [22]; therefore, we did not include these other programs in our study. It is worth noting that the authors of this study concluded that modeling nanobodies remains a big challenge [13].

#### 3.2.6. Nanonet

Nanonet is a deep learning model based on a convolutional neural network [12]. This model uses an algorithm similar to trRosetta, but whose input is the one-hot encoded sequence instead of residue representations, so it does not require an MSA. Nanonet generates only the 3D structure of the protein backbone, so it requires an external tool for modeling the side chains (for this study, we used Modeller [59]). Unlike the rest of the models analyzed in this study, Nanonet seems to be the simplest model theoretically. In this sense, the model does not use representations of sequence residues, either from a comprehensive protein language model, such as ESM-2, OmegaPLM, or AntiBERTy, or extracted from MSA, likeAlphaFold2, and Yang-Server. Because of its simplicity, this model provides a great advantage in terms of time and computational resources. Nanonet was trained with about 1800 non-redundant Nbs and mAb heavy chain structures. Interestingly, Nanonet achieves good accuracy even with the simplest architecture and training data set.

### 3.3. Performance Evaluation Metrics

#### 3.3.1. Structural Similarity Metrics

We used TM-score (template modeling score) [60], GDT_TS (global distance test—total score) [61], and GDT_HA (global distance test—high accuracy) [61] to evaluate the overall modeling accuracy of the different AI models. Both TM-score and GDT measure the structural similarity between two protein structures. GDT is commonly used to compare models with their corresponding crystallographic structures, being the major assessment criterion in the CASP event [51]. The Zhang group’s TM-score program was used to compute the structural alignment, TM, and GDT scores [36,60]. For region-level analysis, we used RMSD (root-mean-square deviation). The RMSD for Cα and all heavy atoms were computed using a ParmEd-based script [62].

#### 3.3.2. Statistics

To estimate the differences between the metrics used in this study, we performed a Kruskal–Wallis one-way analysis of variance. In significant cases, we used Dunn’s test with the Benjamini-Hochberg correction as a *post hoc* test. Dunn’s test is the appropriate nonparametric pairwise multiple comparison procedure when a Kruskal–Wallis test is rejected. Calculations were performed using the bioinfokit tool (v 1.0.5) [63].

#### 3.3.3. Execution Environment

Calculations were performed using low-end and mid-range hardware (AMD Ryzen 7 3700 and a GPU Nvidia 1660 Super 6GB VRAM and 16 GB RAM). All programs were installed in a standalone Miniconda environment with Python 3.8.13, following the instructions given by their developers.

#### 3.3.4. Energy Minimization

For energy minimization, we used OpenMM v7.7.0 [64] with the Amber99SB [65] force field as described in the AlphaFold2 study [31]. The simulation was carried out for a maximum of 50,000 steps and a tolerance of 1000.0 kJ/mol/nm.

## 4. Conclusions

Nowadays, computer-assisted methods have become essential components in protein engineering, especially for antibodies and nanobodies. Whether for structure characterization, antigen interaction, or affinity enhancement through an in silico affinity maturation process, having reliable structural models is extremely important. Using a poor-quality model can lead to erroneous conclusions and low efficiency in further experimental development.

Multiple studies have shown the superiority of AI programs over conventional homology modeling approaches for modeling protein structures. In this study, we have evaluated the performance of six state-of-the-art AI programs in modeling Nb structures. To this aim, we generated a test dataset containing 75 unique Nbs not included in the training sets of the evaluated programs. The performance of different models was assessed using global metrics, as well as metrics for different regions within the structure. The results show that global metrics such as TM-score, GDT-TS, and GDT-HA are unsuitable for Nb structural model evaluation since the modeling errors of highly variable, but functionally important regions such as CDR3 get diluted when using these metrics. We then evaluated the modeling accuracy separately for the framework and CDR regions. OmegaFold achieved the best results, followed by AlphaFold2, ESMFold, IgFold, Nanonet, and Yang-Server.

Although the evaluated AI models represent a leap forward in Nb modeling, they are still far from providing completely reliable structural models. This study confirms that, while modeling of the framework region is consistently good in all cases, CDR modeling remains a challenge, especially for CDR3. For this loop, the RMSDs of the generated models are in most cases considerably high compared to the crystallographic structures. Although the median RMSD is relatively low for all AI models, only 52, 44, 35, 29, 25, and 15% of the CDR3 structures generated with OmegaFold, AlphaFold2, ESMFold, IgFold, Nanonet, and Yang-Server, respectively, were modeled with less than 2.5 Å difference compared to the crystallographic structures. Energy minimization did not improve the results. Since CDR3 is extremely important for antigen interaction, the obtained models may not be suitable for applications that require high accuracy, such as protein–protein docking.

Modeling with these AI programs can be performed using hardware in the low- to mid-range, which facilitates their use in common bioinformatics laboratories. In these conditions, the calculation times vary from a few to hundreds of seconds. Nanonet is the fastest model, followed by IgFold, OmegaFold, and lastly, AlphaFold2, while ESMFold and Yang-Server can be used on their dedicated servers. According to our results, OmegaFold is the most efficient AI program for Nb modeling, being relatively fast and achieving the best results. Similarly, both ESMFold and AlphaFold2 may be used as an alternative, yielding quite similar results compared to OmegaFold.

The Inherent limitations of this type of study must also be considered. The rapid development of artificial intelligence methods for protein modeling makes it almost impossible to keep the evaluation of their performance up to date in real time. In addition, the time lag between the selection of training sets and the release of the programs can lead to biases in a benchmarking study. In this study, we selected a set of nanobodies not included in the training sets of the evaluated programs. This restriction, also associated with the limited number of crystallographic structures available, reduces the representativeness of the evaluation set. Currently, several dozens of AI programs have been developed for protein modeling, as evidenced by the recent CASP15 competition (2022) that included more than twenty AI methods. For this study, we chose to evaluate six of these programs, based on their performance in CASP15, their reported applications (popularity), and accessibility. Therefore, the Nb modeling capabilities of the other AI methods have not yet been evaluated.

So far, although there have been substantial advances, the accuracy of the generated models is still limited and Nb modeling remains a challenge. However, the fast development and improvement of AI models, along with the increase of available crystallographic structures, augur significant advancements in Nb modeling in the near future.

## Data Availability

All files and tools to reproduce the results and analyses can be found at https://github.com/Valdes-Tresanco-MS/NbModelingBenchmark (accessed on 1 May 2023).

## Supplementary Materials

The following supporting information can be downloaded at: https://www.mdpi.com/article/10.3390/molecules28103991/s1, Figure S1. Resolution distribution of crystallographic structures of Nbs in the dataset. Figure S2. Sequence identity among Nbs in the dataset. Figure S3. Distribution of heavy atoms RMSD values by position for the OmegaFold, AlphaFold2, IgFold, and Nanonet models. Figure S4. Representation by positions of the Nbs in the dataset using the Aho numbering scheme. Figure S5. CDR3 length distribution of non-redundant Nbs structures in the Protein Data Bank. Figure S6. Particular cases of structural variations in the crystallographic structures are used as references. Figure S7. Correlation between the RMSD values and the average predicted confidences by AI models for the CDR regions in the 75 Nbs conforming the dataset. Figure S8. Comparison of the RMSD distribution for the models obtained varying the number of recycles of AlphaFold2 and OmegaFold. Figure S9. Structural variation of CDR3 of the same Nb in the asymmetric unit. Figure S10. Comparison between the RMSD of models generated with AlphaFold2 (AF2) and AlphaFold-multimer (AF2m) clustered by CDR3 length. Figure S11. Comparison of the RMSD distribution between the default generated model and its minimized version for OmegaFold, AlphaFold2, IgFold, Nanonet, ESMFold and Yang-server. Table S1. Nbs included in the dataset. Table S2. Results from Dunn’s test nonparametric pairwise multiple comparison procedure. Table S3. Number of occurrences of predicted structures with RMSD above 2.5 Å. Table S4. Correlation between the RMSD values and the average predicted confidences by AI models for the CDR regions in the 75 Nbs conforming the dataset. Table S5. Nanobodies selected for modeling Nb-antigen complexes. All PDB files can be found at https://github.com/Valdes-Tresanco-MS/NbModelingBenchmark (accessed on 1 May 2023).

## References

[1] Hamers-Casterman C., Atarhouch T., Muyldermans S., Robinson G., Hammers C., Songa E.B., Bendahman N., Hammers R. (1993). Naturally Occurring Antibodies Devoid of Light Chains. Nature.

[2] Steeland S., Vandenbroucke R.E., Libert C. (2016). Nanobodies as Therapeutics: Big Opportunities for Small Antibodies. Drug Discov. Today.

[3] Gonzalez-Sapienza G., Rossotti M.A., Tabares-da Rosa S. (2017). Single-Domain Antibodies as Versatile Affinity Reagents for Analytical and Diagnostic Applications. Front. Immunol..

[4] Zare H., Aghamollaei H., Hosseindokht M., Heiat M., Razei A., Bakherad H. (2021). Nanobodies, the Potent Agents to Detect and Treat the Coronavirus Infections: A Systematic Review. Mol. Cell. Probes.

[5] Muyldermans S. (2021). Applications of Nanobodies. Annu. Rev. Anim. Biosci..

[6] Yang E.Y., Shah K. (2020). Nanobodies: Next Generation of Cancer Diagnostics and Therapeutics. Front. Oncol..

[7] Wang J., Kang G., Yuan H., Cao X., Huang H., de Marco A. (2022). Research Progress and Applications of Multivalent, Multispecific and Modified Nanobodies for Disease Treatment. Front. Immunol..

[8] Njeru F.N., Kusolwa P.M. (2021). Nanobodies: Their Potential for Applications in Biotechnology, Diagnosis and Antiviral Properties in Africa; Focus on Application in Agriculture. Biotechnol. Biotechnol. Equip..

[9] Wang X., Chen Q., Sun Z., Wang Y., Su B., Zhang C., Cao H., Liu X. (2020). Nanobody Affinity Improvement: Directed Evolution of the Anti-Ochratoxin a Single Domain Antibody. Int. J. Biol. Macromol.

[10] Soler M.A., Fortuna S., de Marco A., Laio A. (2018). Binding Affinity Prediction of Nanobody-Protein Complexes by Scoring of Molecular Dynamics Trajectories. Phys. Chem. Chem. Phys..

[11] Hacisuleyman A., Erman B. (2019). ModiBodies: A Computational Method for Modifying Nanobodies to Improve Their Antigen Binding Affinity and Specificity. bioRxiv.

[12] Cohen T., Halfon M., Schneidman-Duhovny D. (2022). NanoNet: Rapid and Accurate End-to-End Nanobody Modeling by Deep Learning. Front. Immunol..

[13] Ruffolo J.A., Chu L.-S., Mahajan S.P., Gray J.J. (2022). Fast, Accurate Antibody Structure Prediction from Deep Learning on Massive Set of Natural Antibodies. bioRxiv.

[14] Berman H.M., Westbrook J., Feng Z., Gilliland G., Bhat T.N., Weissig H., Shindyalov I.N., Bourne P.E. (2000). The Protein Data Bank. Nucleic Acids Res..

[15] Burley S.K., Berman H.M., Bhikadiya C., Bi C., Chen L., di Costanzo L., Christie C., Dalenberg K., Duarte J.M., Dutta S. (2019). RCSB Protein Data Bank: Biological Macromolecular Structures Enabling Research and Education in Fundamental Biology, Biomedicine, Biotechnology and Energy. Nucleic Acids Res..

[16] Mitchell L.S., Colwell L.J. (2018). Analysis of Nanobody Paratopes Reveals Greater Diversity than Classical Antibodies. Protein Eng. Des. Sel..

[17] Xi X., Sun W., Su H., Zhang X., Sun F. (2020). Identification of a Novel Anti-EGFR Nanobody by Phage Display and Its Distinct Paratope and Epitope via Homology Modeling and Molecular Docking. Mol. Immunol..

[18] Cheng X., Wang J., Kang G., Hu M., Yuan B., Zhang Y., Huang H. (2019). Homology Modeling-Based in Silico Affinity Maturation Improves the Affinity of a Nanobody. Int. J. Mol. Sci..

[19] Zou J. (2022). Artificial Intelligence Revolution in Structure Prediction for Entire Proteomes. MedComm—Future Med..

[20] Bertoline L.M.F., Lima A.N., Krieger J.E., Teixeira S.K. (2023). Before and after AlphaFold2: An Overview of Protein Structure Prediction. Front. Bioinform..

[21] Tunyasuvunakool K. (2022). The Prospects and Opportunities of Protein Structure Prediction with AI. Nat. Rev. Mol. Cell Biol..

[22] Abanades B., Georges G., Bujotzek A., Deane C.M. (2022). ABlooper: Fast Accurate Antibody CDR Loop Structure Prediction with Accuracy Estimation. Bioinformatics.

[23] Ruffolo J.A., Gray J.J., Sulam J. (2021). Deciphering Antibody Affinity Maturation with Language Models and Weakly Supervised Learning. arXiv.

[24] Wang S., Sun S., Li Z., Zhang R., Xu J. (2017). Accurate De Novo Prediction of Protein Contact Map by Ultra-Deep Learning Model. PLoS Comput. Biol..

[25] Fernández-Quintero M.L., Kokot J., Waibl F., Fischer A.L.M., Quoika P.K., Deane C.M., Liedl K.R. (2023). Challenges in Antibody Structure Prediction. MAbs.

[26] Ruffolo J.A., Sulam J., Gray J.J. (2022). Antibody Structure Prediction Using Interpretable Deep Learning. Patterns.

[27] Ruffolo J.A., Guerra C., Mahajan S.P., Sulam J., Gray J.J. (2020). Geometric Potentials from Deep Learning Improve Prediction of CDR H3 Loop Structures. Bioinformatics.

[28] AlQuraishi M. (2021). Machine Learning in Protein Structure Prediction. Curr. Opin. Chem. Biol..

[29] Eisenstein M. (2021). Artificial Intelligence Powers Protein-Folding Predictions. Nature.

[30] AlQuraishi M. (2021). Protein-Structure Prediction Revolutionized. Nature.

[31] Jumper J., Evans R., Pritzel A., Green T., Figurnov M., Ronneberger O., Tunyasuvunakool K., Bates R., Žídek A., Potapenko A. (2021). Highly Accurate Protein Structure Prediction with AlphaFold. Nature.

[32] Callaway E. (2022). What’s next for AlphaFold and the AI Protein-Folding Revolution. Nature.

[33] Wu R., Ding F., Wang R., Shen R., Zhang X., Luo S., Su C., Wu Z., Xie Q., Berger B. (2022). High-Resolution de Novo Structure Prediction from Primary Sequence. bioRxiv.

[34] Lin Z., Akin H., Rao R., Hie B., Zhu Z., Lu W., Smetanin N., Verkuil R., Kabeli O., Shmueli Y. (2022). Evolutionary-Scale Prediction of Atomic Level Protein Structure with a Language Model. bioRxiv.

[35] Schoof M., Faust B., Saunders R.A., Sangwan S., Rezelj V., Hoppe N., Boone M., Billesbølle C.B., Puchades C., Azumaya C.M. (2020). An Ultrapotent Synthetic Nanobody Neutralizes SARS-CoV-2 by Stabilizing Inactive Spike. Science.

[36] Xu J., Zhang Y. (2010). How Significant Is a Protein Structure Similarity with TM-Score = 0.5?. Bioinformatics.

[37] Du Z., Su H., Wang W., Ye L., Wei H., Peng Z., Anishchenko I., Baker D., Yang J. (2021). The TrRosetta Server for Fast and Accurate Protein Structure Prediction. Nat. Protoc..

[38] Hong J., Kwon H.J., Cachau R., Chen C.Z., Butay K.J., Duan Z., Li D., Ren H., Liang T., Zhu J. (2021). Camel Nanobodies Broadly Neutralize SARS-CoV-2 Variants. bioRxiv.

[39] Frosi Y., Lin Y.C., Shimin J., Ramlan S.R., Hew K., Engman A.H., Pillai A., Yeung K., Cheng Y.X., Cornvik T. (2022). Engineering an Autonomous VH Domain to Modulate Intracellular Pathways and to Interrogate the EIF4F Complex. Nat. Commun..

[40] Mirdita M., Schütze K., Moriwaki Y., Heo L., Ovchinnikov S., Steinegger M. (2022). ColabFold: Making Protein Folding Accessible to All. Nat. Methods.

[41] Evans R., O’Neill M., Pritzel A., Antropova N., Senior A., Green T., Žídek A., Bates R., Blackwell S., Yim J. (2022). Protein Complex Prediction with AlphaFold-Multimer. bioRxiv.

[42] Zimmermann I., Egloff P., Hutter C.A.J., Arnold F.M., Stohler P., Bocquet N., Hug M.N., Hub er S., Siegrist M., Hetemann L. (2018). Synthetic Single Domain Antibodies for the Conformational Trapping of Membrane Proteins. Elife.

[43] Moreno E., Valdés-Tresanco M.S., Molina-Zapata A., Sánchez-Ramos O. (2022). Structure-Based Design and Construction of a Synthetic Phage Display Nanobody Library. BMC Res. Notes.

[44] Valdés-Tresanco M.S., Molina-Zapata A., Pose A.G., Moreno E. (2022). Structural Insights into the Design of Synthetic Nanobody Libraries. Molecules.

[45] Dunbar J., Krawczyk K., Leem J., Baker T., Fuchs A., Georges G., Shi J., Deane C.M. (2014). SAbDab: The Structural Antibody Database. Nucleic Acids Res..

[46] Altschul S.F., Gish W., Miller W., Myers E.W., Lipman D.J. (1990). Basic Local Alignment Search Tool. J. Mol. Biol..

[47] Camacho C., Coulouris G., Avagyan V., Ma N., Papadopoulos J., Bealer K., Madden T.L. (2009). BLAST+: Architecture and Applications. BMC Bioinform..

[48] Altschul S.F., Madden T.L., Schäffer A.A., Zhang J., Zhang Z., Miller W., Lipman D.J. (1997). Gapped BLAST and PSI-BLAST: A New Generation of Protein Database Search Programs. Nucleic Acids Res..

[49] Dunbar J., Deane C.M. (2016). ANARCI: Antigen Receptor Numbering and Receptor Classification. Bioinformatics.

[50] Yang J., Anishchenko I., Park H., Peng Z., Ovchinnikov S., Baker D. (2020). Improved Protein Structure Prediction Using Predicted Interresidue Orientations. Proc. Natl. Acad. Sci. USA.

[51] Pereira J., Simpkin A.J., Hartmann M.D., Rigden D.J., Keegan R.M., Lupas A.N. (2021). High-Accuracy Protein Structure Prediction in CASP14. Proteins.

[52] Mirdita M., Steinegger M., Söding J. (2019). MMseqs2 Desktop and Local Web Server App for Fast, Interactive Sequence Searches. Bioinformatics.

[53] Steinegger M., Söding J. (2017). MMseqs2 Enables Sensitive Protein Sequence Searching for the Analysis of Massive Data Sets. Nat. Biotechnol..

[54] Rives A., Meier J., Sercu T., Goyal S., Lin Z., Liu J., Guo D., Ott M., Zitnick C.L., Ma J. (2021). Biol.ogical Structure and Function Emerge from Scaling Unsupervised Learning to 250 Million Protein Sequences. Proc. Natl. Acad. Sci. USA.

[55] Suzek B.E., Huang H., McGarvey P., Mazumder R., Wu C.H. (2007). UniRef: Comprehensive and non-redundant UniProt reference clusters. Bioinformatics.

[56] Evans R., Jumper J., Kirkpatrick J., Sifre L., Green T.F.G., Qin C., Zidek A., Nelson A., Bridgland A., Penedones H. De Novo Structure Prediction with Deep-Learning Based Scoring. Proceedings of the Thirteenth Critical Assessment of Techniques for Protein Structure Prediction (ProteinStructure Prediction Center).

[57] Dunbar J., Krawczyk K., Leem J., Marks C., Nowak J., Regep C., Georges G., Kelm S., Popovic B., Deane C.M. (2016). SAbPred: A Structure-Based Antibody Prediction Server. Nucleic Acids Res..

[58] Chaudhury S., Lyskov S., Gray J.J. (2010). PyRosetta: A Script-Based Interface for Implementing Molecular Modeling Algorithms Using Rosetta. Bioinformatics.

[59] Šali A., Blundell T.L. (1993). Comparative protein modelling by satisfaction of spatial restraints. J. Mol. Biol..

[60] Zhang Y., Skolnick J. (2004). Scoring Function for Automated Assessment of Protein Structure Template Quality. Proteins.

[61] Zemla A. (2003). LGA: A Method for Finding 3D Similarities in Protein Structures. Nucleic Acids Res..

[62] Shirts M.R., Klein C., Swails J.M., Yin J., Gilson M.K., Mobley D.L., Case D.A., Zhong E.D. (2016). Lessons Learned from Comparing Molecular Dynamics Engines on the SAMPL5 Dataset. J. Comput. Aided Mol. Des..

[63] Bedre R. (2021). Bioinfokit: Bioinformatics Data Analysis and Visualization Toolkit, version 1.0.5.

[64] Eastman P., Swails J., Chodera J.D., McGibbon R.T., Zhao Y., Beauchamp K.A., Wang L.P., Simmonett A.C., Harrigan M.P., Stern C.D. (2017). OpenMM 7: Rapid Development of High Performance Algorithms for Molecular Dynamics. PLoS Comput. Biol..

[65] Hornak V., Abel R., Okur A., Strockbine B., Roitberg A., Simmerling C. (2006). Comparison of Multiple Amber Force Fields and Development of Improved Protein Backbone Parameters. Proteins Struct. Funct. Bioinform..

