# Supplementary material for: Structural Modeling of Nanobodies: A Benchmark of State-of-the-Art Artificial Intelligence Programs
Source: Molecules. 2023 May 9;28(10):3991. doi: 10.3390/molecules28103991 (PMC10220908; doi:10.3390/molecules28103991)
Supplement: Supplementary file 1 [file molecules-28-03991-s001.zip › molecules-2362608-supplementary.pdf]

Structural modeling of nanobodies: A benchmark of state-of-the-art artificial intelligence programs

Mario S. Valdés-Tresanco 1,*,†, Mario E. Valdés-Tresanco 2,†, Daiver E. Jiménez-Gutiérrez 1 and Ernesto Moreno 1,*

1 Faculty of Basic Sciences, University of Medellin, Medellin 050026, Colombia; daiverjimenez05@gmail.com

2 Centre for Molecular Simulations and Department of Biological Sciences, University of Calgary, Calgary, AB T2N 1N4, Canada; mario.valdeztresanco@ucalgary.ca

* Correspondence: mariosergiovaldes145@gmail.com (M.S.V.-T.); emoreno@udemedellin.edu.co (E.M.)

† These authors contributed equally to this work.

Table S1. Nbs included in the dataset. All PDB files can be found at: https://github.com/Valdes-Tresanco-MS/NbModelingBenchmark

PDB	PDB	PDB	PDB	PDB
7d8b_B	7p5y_G	7ndf_C	7lzp_B	7rby_B
7anq_B	7czd_A	7d6y_B	7apj_B	7e53_B
7r4r_D	7rnn_C	7fat_B	7nfr_B	7djy_A
7f5g_B	7vfa_D	6zwk_A	7nqa_C	7p5w_G
7r98_D	7s2r_B	7o06_A	7r74_B	7omn_A
7fau_B	7p5v_G	7r4q_D	7nft_C	7t5f_B
7m1h_F	7z1a_E	7r20_B	7lzp_C	7vfb_B
7nfq_C	7pqg_B	7qbg_E	7lzp_G	7olz_B
7p6k_G	7kd0_C	7oan_F	7na9_D	7ocy_C
7e6u_B	7p60_G	7r63_A	7djx_A	7z1c_E
7qiv_C	7l6v_B	7sl9_B	7b2p_D	7tpr_F
7qbe_E	7t5f_C	7php_N	7d4b_B	7r4i_D
7m1h_E	7te8_A	7ooi_A	7zfb_M	7l6v_D
7n9c_D	7nqk_B	7s7r_B	7l6v_C	7ar0_B
7ngh_D	7tpr_D	7cz0_E	7z1b_E	7te8_C

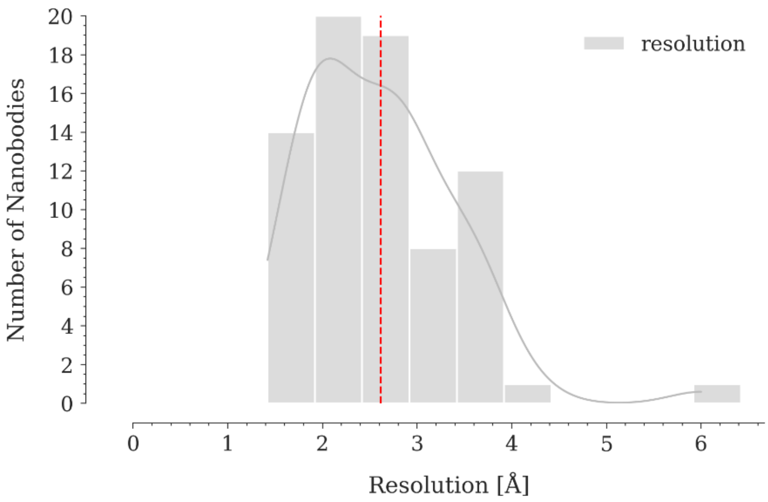

Figure S1. Resolution distribution of crystallographic structures of Nbs in the dataset. The vertical red dashed line represents the median resolution value.

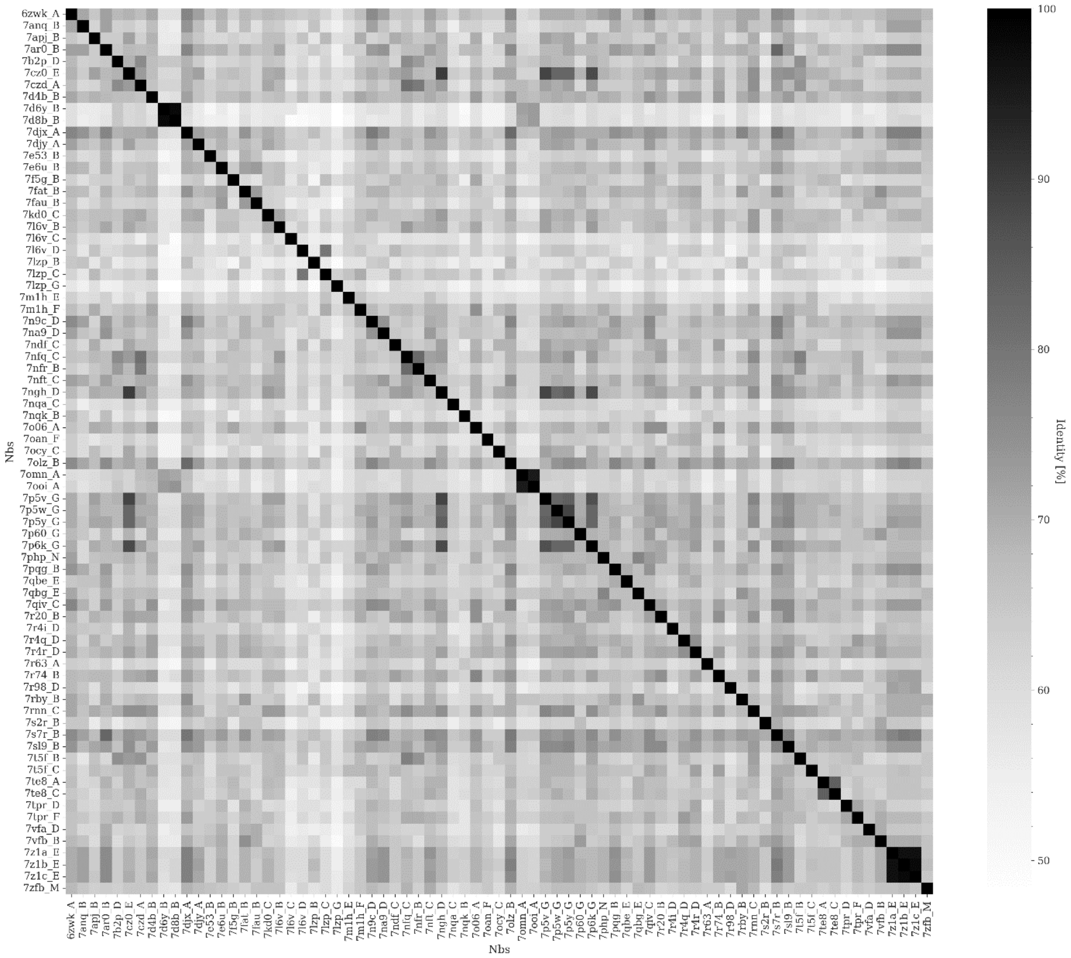

Figure S2. Sequence identity among Nbs in the dataset. Only 4 Nbs have sequence identities greater than 95%.

Table S2. Results from Dunn's test nonparametric pairwise multiple comparison procedure.

Method1	Method2	TM-score	GDT_TS	GDT_HA	Fw	CDR1	CDR2	CDR3	Global
OF	AF2	ns	ns	ns	ns	ns	ns	ns	ns
OF	IF	*	*	*	ns	ns	ns	*	ns
OF	NN	**	**	**	ns	ns	ns	***	**
OF	ESM	ns	ns	ns	ns	ns	ns	ns	ns
OF	YS	****	****	****	****	***	****	****	****
AF2	OF	ns	ns	ns	ns	ns	ns	ns	ns
AF2	IF	ns	ns	ns	ns	ns	ns	ns	ns
AF2	NN	*	*	*	ns	ns	ns	*	ns
AF2	ESM	ns	ns	ns	ns	ns	ns	ns	ns
AF2	YS	****	****	****	****	***	****	***	****
IF	OF	*	*	*	ns	ns	ns	*	ns
IF	AF2	ns	ns	ns	ns	ns	ns	ns	ns
IF	NN	ns	ns	ns	ns	ns	ns	ns	ns
IF	ESM	ns	ns	ns	ns	ns	ns	ns	ns
IF	YS	****	****	****	****	*	***	*	****
NN	OF	**	**	**	ns	ns	ns	***	**
NN	AF2	*	*	*	ns	ns	ns	*	ns
NN	IF	ns	ns	ns	ns	ns	ns	ns	ns
NN	ESM	*	*	*	ns	ns	ns	ns	ns
NN	YS	****	****	****	****	ns	***	ns	****
ESM	OF	ns	ns	ns	ns	ns	ns	ns	ns
ESM	AF2	ns	ns	ns	ns	ns	ns	ns	ns
ESM	IF	ns	ns	ns	ns	ns	ns	ns	ns
ESM	NN	*	*	*	ns	ns	ns	ns	ns
ESM	YS	****	****	****	****	***	****	***	****
YS	OF	****	****	****	****	***	****	****	****
YS	AF2	****	****	****	****	***	****	***	****
YS	IF	****	****	****	****	*	***	*	****
YS	NN	****	****	****	****	ns	***	ns	****
YS	ESM	****	****	****	****	***	****	***	****

Table S3. Number of occurrences of predicted structures with RMSD above 2.5 Å.

Model	CDR1	CDR2	CDR3	Fw	Global
OF	18	4	36		8
AF2	18	4	42		10
IF	18	6	53		9
NN	20	3	56		14
ESM	20	3	49		11
YS	28	7	64	1	28

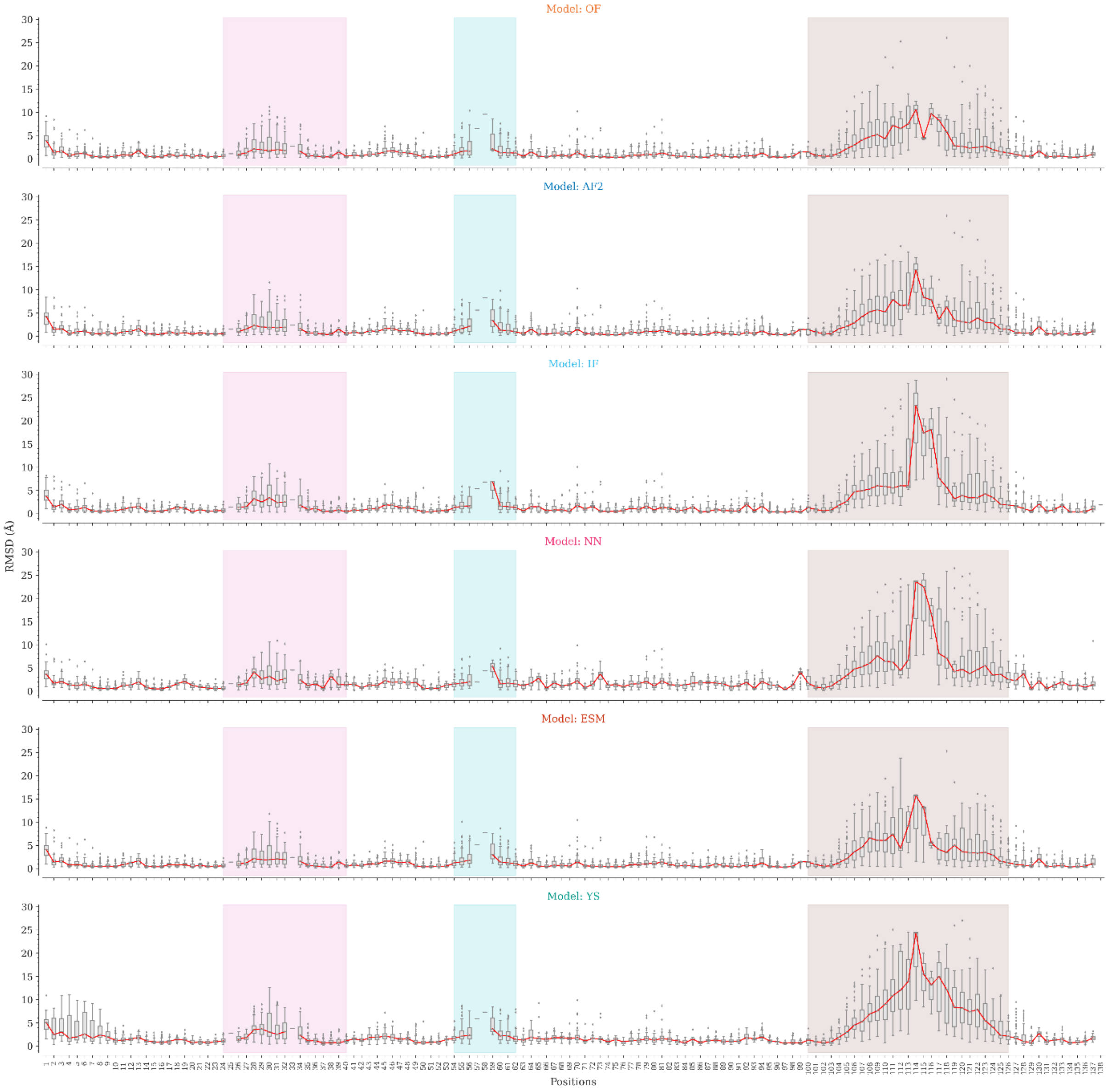

Figure S3. Distribution of heavy atoms RMSD values by position for the OmegaFold, AlphaFold2, IgFold, and Nanonet models. CDR1, CDR2, and CDR3 regions are colored pink, cyan, and brown, respectively. The RMSD distributions are represented by a boxplot. The lower and upper edges of the box represent the first (Q1) and third quartile (Q3), respectively. The difference Q3 - Q1 is known as the interquartile range (IQR). Whiskers extend to the minimum and maximum points within ±1.5 × IQR, respectively. The red line indicates the median value by position.

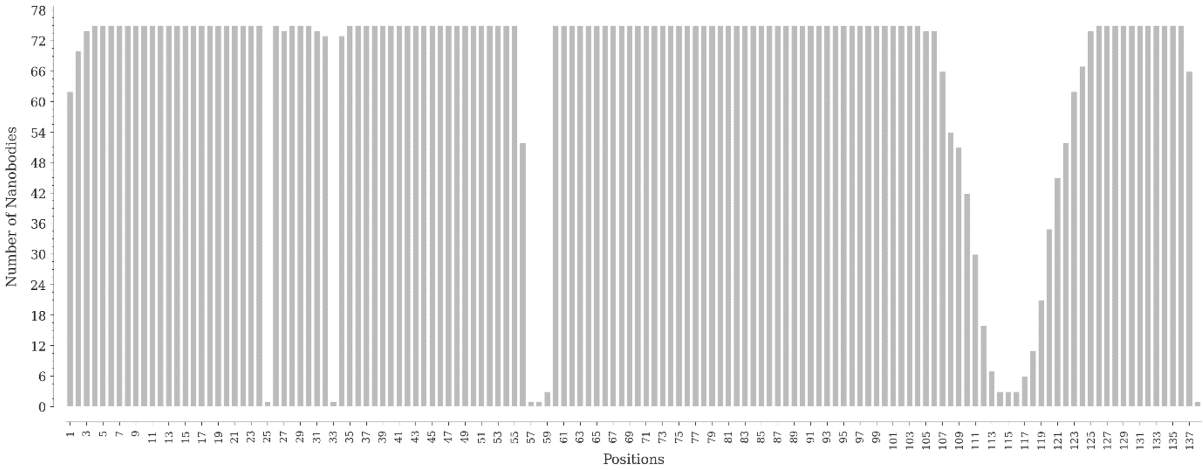

Figure S4. Representation by positions of the Nbs in the dataset using the Aho numbering scheme.

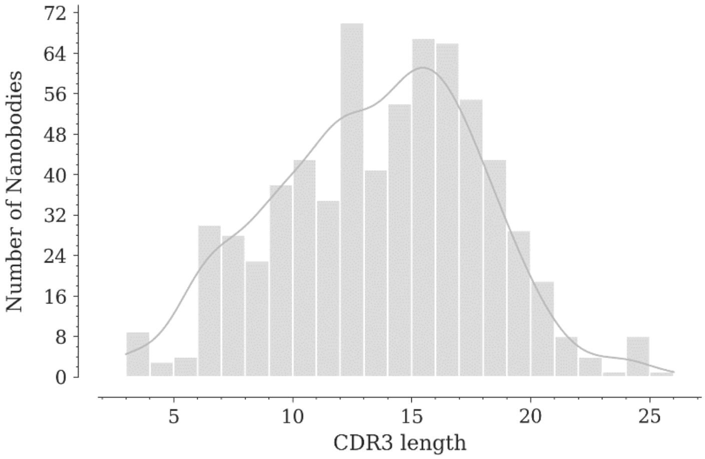

Figure S5. CDR3 length distribution of non-redundant Nbs structures in the Protein Data Bank.

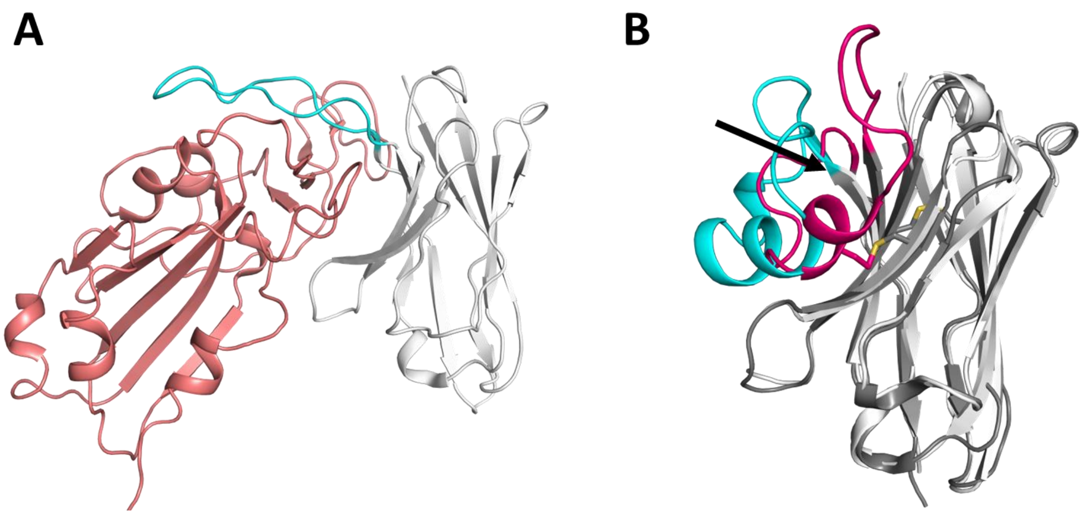

Figure S6. Particular cases of structural variations in the crystallographic structures are used as references. A) PDB: 7tpr. Nb (white) in complex with the antigen (deep salmon), which sits below CDR3 (cyan) stretching it away from the framework. B) Comparison between the engineered human heavy chain variable domain (PDB: 7db8) without canonical disulfide bridge (framework in white and CDR3 in cyan) and a Nb of equal length (PDB: 5lhn) with canonical disulfide bridge (framework in dark gray and CDR3 in hot pink). The arrow indicates the displacement of the N-terminal portion of the VH CDR3 with respect to the Nb.

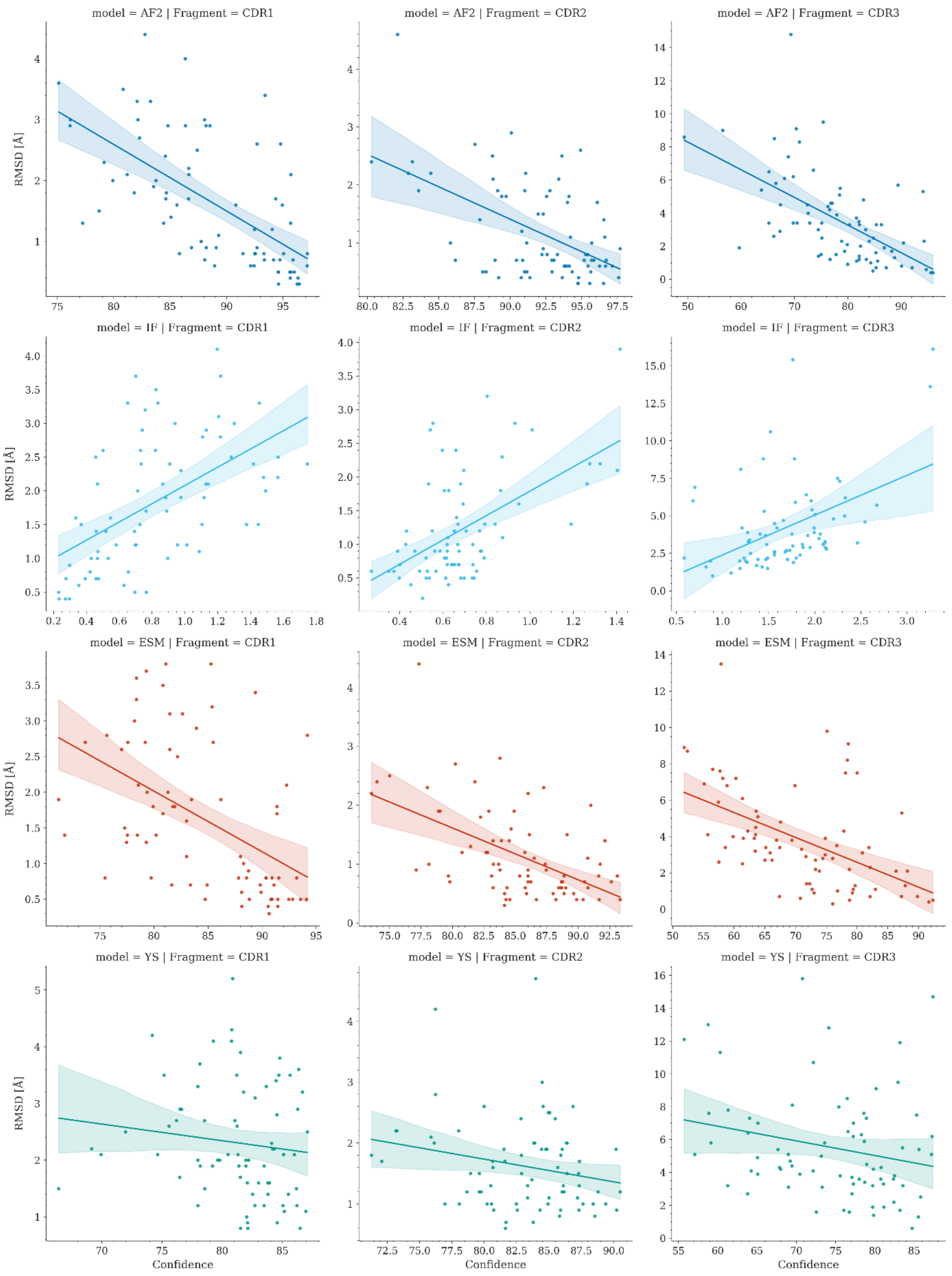

Figure S7. Correlation between the RMSD values and the average predicted confidences by AI models for the CDR regions in the 75 Nbs conforming the dataset. Regression lines relating the RMSD and the average predicted confidence variables are shown in different colors depending on the AI model used. Translucent bands

around the regression lines indicate the 95% confidence interval for the regression estimates.

Table S4. Correlation between the RMSD values and the average predicted confidences by AI models for the CDR regions in the 75 Nbs conforming the dataset.

Model	Region	Pearson	Pearson p-value	Spearman	Spearman p-value	Slope	Intercept
AF2	CDR1	-0.62	2.10E-09	-0.68	1.49E-11	-0.11	11.34
AF2	CDR2	-0.54	7.46E-07	-0.42	2.12E-04	-0.11	11.50
AF2	CDR3	-0.59	3.02E-08	-0.63	1.14E-09	-0.17	16.65
OF	CDR1	-0.59	2.57E-08	-0.62	3.55E-09	-0.08	8.68
OF	CDR2	-0.44	6.55E-05	-0.47	2.08E-05	-0.07	7.44
OF	CDR3	-0.62	4.01E-09	-0.71	7.02E-13	-0.13	12.87
IF	CDR1	0.54	5.91E-07	0.60	1.42E-08	1.35	0.73
IF	CDR2	0.55	3.13E-07	0.47	2.21E-05	1.81	-0.02
IF	CDR3	0.46	3.61E-05	0.45	4.86E-05	2.65	-0.26
ESM	CDR1	-0.48	1.05E-05	-0.54	7.34E-07	-0.08	8.81
ESM	CDR2	-0.54	6.66E-07	-0.49	7.73E-06	-0.09	8.66
ESM	CDR3	-0.51	2.45E-06	-0.56	2.23E-07	-0.14	13.52
YS	CDR1	-0.13	2.81E-01	-0.21	7.02E-02	-0.03	4.69
YS	CDR2	-0.22	5.91E-02	-0.20	8.72E-02	-0.04	4.72
YS	CDR3	-0.23	5.01E-02	-0.24	3.55E-02	-0.09	12.18

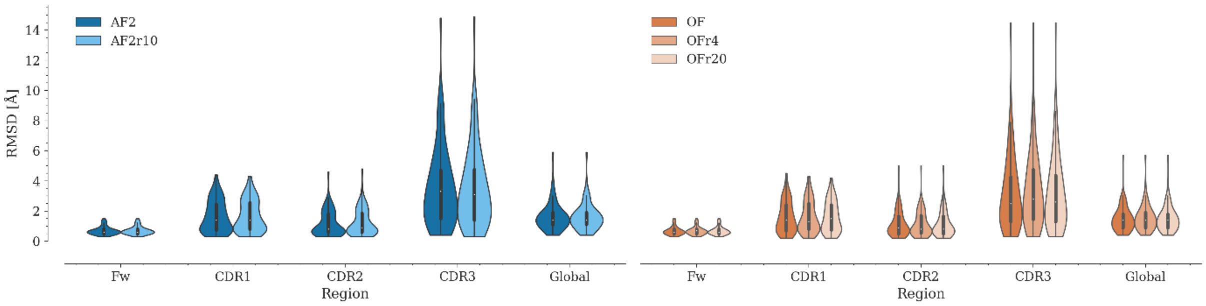

Figure S8. Comparison of the RMSD distribution for the models obtained varying the number of recycles of AlphaFold2 and OmegaFold. The RMSD distributions are represented by a violin plot. It combines the kernel density plot (outer) to show the distribution of values and a boxplot (inner) that summarizes the distribution statistics. The boxplot is composed of the white dot representing the median, the thick gray bar in the center representing the interquartile range, and the thin gray line representing the rest of the distribution. No statistically significant differences were found between the models obtained with the default parameters and those obtained with varying the number of recycles of AlphaFold2 and OmegaFold.

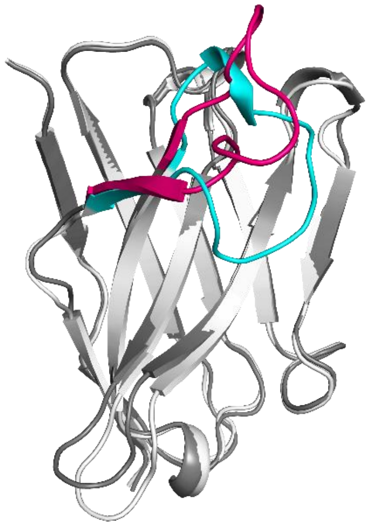

Figure S9. Structural variation of CDR3 of the same Nb in the asymmetric unit. Both CDR3 are highlighted in cyan and hotpink, respectively. PDB ID: 7omn

Table S5. Nanobodies selected for modeling Nb-antigen complexes. All PDB files can be found at https://github.com/Valdes-Tresanco-MS/NbModelingBenchmark

PDB	PDB	PDB	PDB	PDB
7d8b_B	7l6v_D	7ocy_C	7m1h_F	7z1b_E
7nft_C	7rby_B	7d4b_B	7vfa_D	7fau_B
7nfr_B	7lzp_C	7l6v_B	7php_N	7anq_B
7m1h_E	7z1c_E	7olz_B	7d6y_B	7fat_B
7sl9_B	7o06_A	7ar0_B	7qbg_E	7qbe_E
7t5f_B	7lzp_B	7na9_D	7s2r_B	7r74_B
7r98_D	7e53_B	7f5g_B	7zfb_M	7ndf_C
7t5f_C	7ngh_D	7rnn_C	7lzp_G	7nfq_C
7pqg_B				

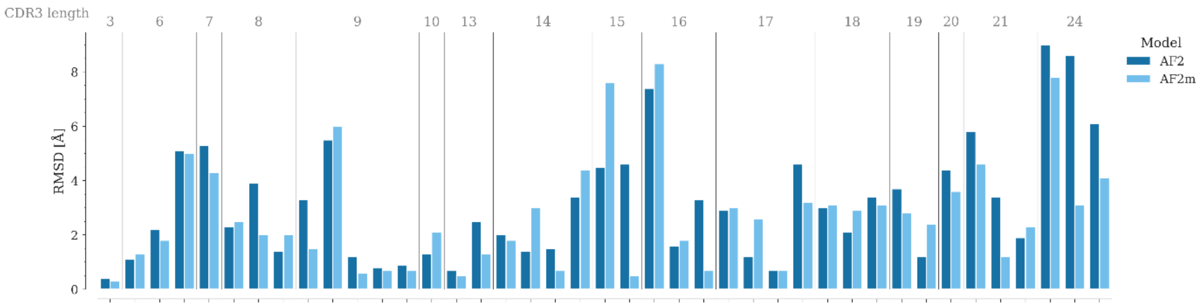

Figure S10. Comparison between the RMSD of models generated with AlphaFold2 (AF2) and AlphaFold-multimer (AF2m) clustered by CDR3 length.

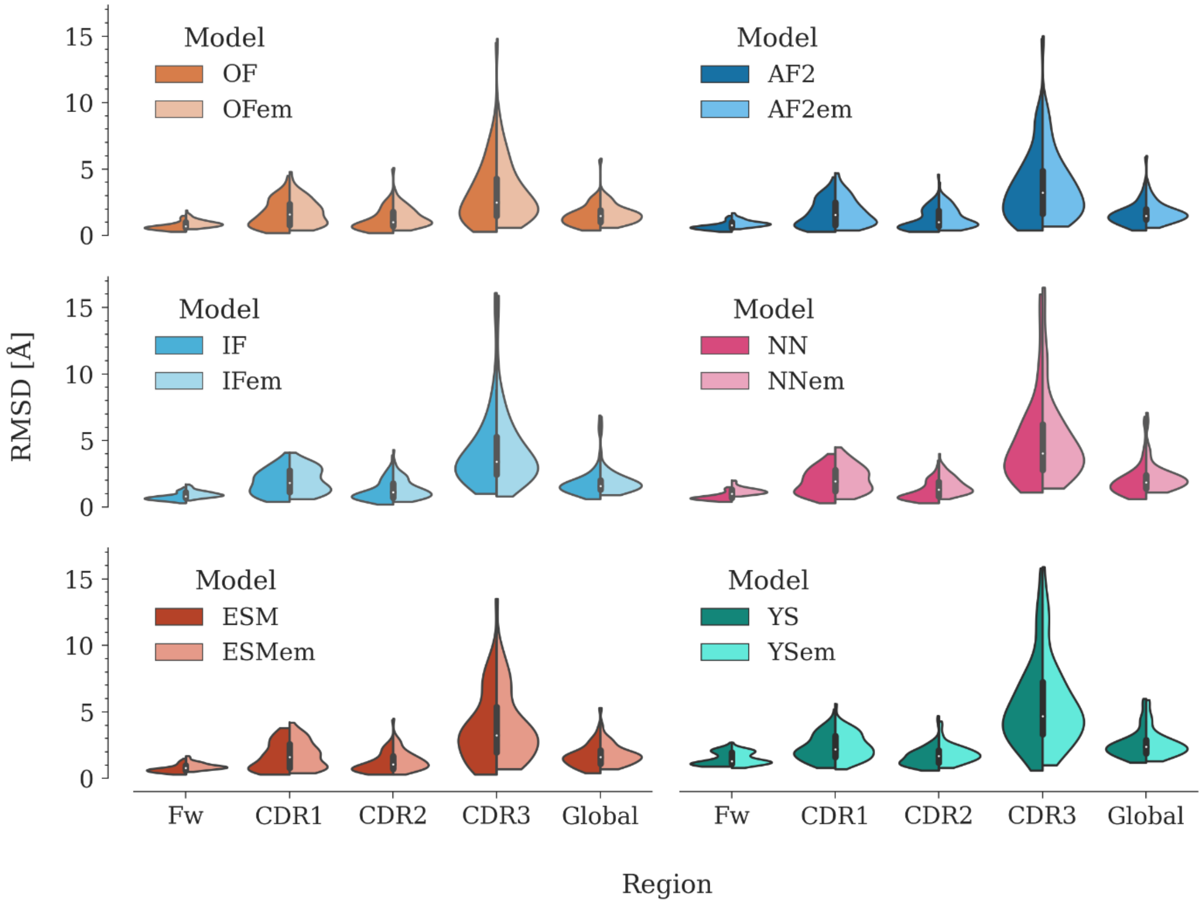

Figure S11. Comparison of the RMSD distribution between the default generated model and its minimized version for OmegaFold, AlphaFold2, IgFold, Nanonet, ESMFold and Yang-server. The RMSD distributions are represented by a violin plot. It combines the kernel density plot (outer) to show the distribution of values and a boxplot (inner) that summarizes the distribution statistics. The boxplot is composed of the white dot representing the median, the thick gray bar in the center representing the interquartile range, and the thin gray line representing the rest of the distribution. No statistically significant differences were found between the models obtained with the default parameters and their minimized version.
